# Inherited ichthyoses: molecular causes of the disease in Czech patients

**DOI:** 10.1186/s13023-019-1076-7

**Published:** 2019-05-02

**Authors:** Romana Borská, Blanka Pinková, Kamila Réblová, Hana Bučková, Lenka Kopečková, Jitka Němečková, Alena Puchmajerová, Marcela Malíková, Markéta Hermanová, Lenka Fajkusová

**Affiliations:** 1 Centre of Molecular Biology and Gene Therapy, University Hospital Brno and Masaryk University, Jihlavská 20, 625 00 Brno, Czech Republic; 2 Department of Pediatric Dermatology, Pediatric Clinic, University Hospital Brno and Masaryk University, Jihlavská 20, 625 00 Brno, Czech Republic; 3 Central European Institute of Technology, Masaryk University, Kamenice 753/5, 625 00 Brno, Czech Republic; 4 Department of Medical Genetics, University Hospital Brno, Jihlavská 20, 625 00 Brno, Czech Republic; 5 Department of Public Health, Faculty of Medicine, Masaryk University, Kamenice 5, 625 00 Brno, Czech Republic; 6 Institute of Biology and Medical Genetics, University Hospital Motol, V Úvalu 84, 150 06 Prague, Czech Republic; 7 GENNET, Kostelní 9/292, 170 00 Prague, Czech Republic; 8 First Department of Pathological Anatomy, Faculty of Medicine, Masaryk University and St. Anne’s University Hospital, Pekařská 664/53, 656 91 Brno, Czech Republic; 9 Laboratory of Functional Genomics and Proteomics, NCBR, Faculty of Science, Kotlářská 267/2, 611 37 Brno, Czech Republic

**Keywords:** Autosomal recessive congenital ichthyosis, Keratinopathic ichthyosis, In silico analysis, 3D protein structure

## Abstract

Inherited ichthyoses belong to a large and heterogeneous group of mendelian disorders of cornification, and can be distinguished by the quality and distribution of scaling and hyperkeratosis, by other dermatologic and extracutaneous involvement, and by inheritance. We present the genetic analysis results of probands with X-linked ichthyosis, autosomal recessive congenital ichthyosis, keratinopathic ichthyosis, and a patient with Netherton syndrome. Genetic diagnostics was complemented by in silico missense variant analysis based on 3D protein structures and commonly used prediction programs to compare the yields of these two approaches to each other. This analysis revealed various structural defects in proteins coded by mutated genes while no defects were associated with known polymorphisms. Two patients with pathogenic variants in the *ABCA12* gene have a premature termination codon mutation on one allele and a silent variant on the second. The silent variants c.69G > A and c.4977G > A are localised in the last nucleotide of exon 1 and exon 32, respectively, and probably affect mRNA splicing. The phenotype of both patients is very severe, including a picture harlequin foetus after birth; later (at 3 and 6 years of age, respectively) ectropin, eclabion, generalised large polygonal scaling and erythema.

**Electronic supplementary material:**

The online version of this article (10.1186/s13023-019-1076-7) contains supplementary material, which is available to authorized users.

Dear Editor,

Inherited ichthyoses are a heterogeneous group of disorders classified by the quality and distribution of scaling and hyperkeratosis, by other dermatologic and extracutaneous involvement, and by inheritance [1]. The aim of our study was identify variants in genes related to an ichthyosis phenotype and evaluate their pathogenicity. From this reason, we introduced targeted sequencing of 180 genodermatosis related genes (20 of which associated with an ichthyosis – *STS, ABCA12, ALOXE3, ALOX12B, CERS3, CYP4F22, LIPN, NIPAL4, POMP, PNPLA1, TGM1, KRT1, KRT2, KRT10, ABHD5, ALDH3A2, GJB2, SLC27A4, SPINK5, ST14*) and structural analysis of 3D proteins.

Overall, we found 47 probands with autosomal recessive congenital ichthyosis (ARCI), 9 probands with keratinopathic ichthyosis (KI), 2 probands with X-linked ichthyosis (XLI, patients with the *STS* gene deletion are not included), and one proband with Netherton syndrome (Table 1). The patients’ clinical findings are presented in Additional file 1. From 47 ARCI patients, 18 patients (38.3%) have pathogenic sequence variants in *ALOX12B*, 9 patients (19.1%) in *ALOXE3*, 6 patients (12.8%) in *NIPAL4*, 5 patients (10.6%) in *CYP4F22*, 6 patients (12.8%) in *TGM1*, and 3 patients (6.4%) in *ABCA12*. Among analysed patients’ DNA, three atypical sequence changes were identified: 1) a large gene deletion determined by SNP array in patient 27 [p.(Pro630Leu) on the second *ALOXE3* allele]; 2) the variant c.69G > A, p.(Pro23=), r.(spl?) in patient 45 [p.(Arg1881*) on the second *ABCA12* allele]; 3) the variant c.4977G > A, p.(Glu1659=), r.(spl?) in patient 46 [c.483_484delCGinsT on the second *ABCA12* allele]. In silico analyses of c.69G > A (the last nucleotide of exon 1) and c.4977G > A (the last nucleotide of exon 32) using the mutation analysis software Alamut Visual v.2.10.0 revealed that these variants probably affect splicing on the basis of a weakening of the authentic 5′ donor splice sites of exon 1 (− 25.4%) and exon 32 (− 69.4%), respectively.

**Table 1:** Pathogenic sequence variants identified in Czech probands with ichthyosis

No.	Gene	1st allele (cDNA level, protein level)	2nd allele (cDNA level, protein level)
1	*ALOX12B*	c.467_470dupATGT, p.(His158Cysfs*20)	c.1562A > G, p.(Tyr521Cys)
2	*ALOX12B*	c.581A > T, p.(Lys194*)	c.1562A > G, p.(Tyr521Cys)
3	*ALOX12B*	**c.665A > T, p.(Lys222Ile)**	c.1562A > G, p.(Tyr521Cys)
4	*ALOX12B*	**c.787_789delTTC, p.(Phe262del)**	c.1562A > G, p.(Tyr521Cys)
5	*ALOX12B*	c.1034-1035delTT, p.(Phe345Trpfs*28)	c.1790C > A, p.(Ala597Glu)
6	*ALOX12B*	**1071G > C, p.(Gln357His)**	c.1654 + 3A > G, r.(spl?)
7	*ALOX12B*	**c.1156C > T, p.(Arg386Cys)**	c.1654 + 3A > G, r.(spl?)
8	*ALOX12B*	**c.1156C > T, p.(Arg386Cys)**	c.1790C > A, p.(Ala597Glu)
9	*ALOX12B*	**c.1157G > A, p.(Arg386His)**	c.1265C > T, p.(Pro422Leu)
10	*ALOX12B*	**c.1157G > A, p.(Arg386His)**	c.1562A > G, p.(Tyr521Cys)
11	*ALOX12B*	c.1294C > T, p.(Arg432*)	c.1562A > G, p.(Tyr521Cys)
12	*ALOX12B*	**c.1405C > T, p.(Arg469Trp)**	c.1454_1455delTT, p.(Phe485Cysfs*16)
13	*ALOX12B*	**c.1448A > G, p.(Asn483Ser)**	c.1562A > G, p.(Tyr521Cys)
14	*ALOX12B*	**c.1496G > A, p.(Arg499His)**	**c.1496G > A, p.(Arg499His)**
15	*ALOX12B*	c.1562A > G, p.(Tyr521Cys)	**c.1688 T > C, p.(Leu563Pro)**
16	*ALOX12B*	c.1562A > G, p.(Tyr521Cys)	c.1790C > A, p.(Ala597Glu)
17	*ALOX12B*	c.1562A > G, p.(Tyr521Cys)	c.1790C > A, p.(Ala597Glu)
18	*ALOX12B*	c.1918delG, p.(Asp640Thrfs*23)	c.1918delG, p.(Asp640Thrfs*23)
19	*ALOXE3*	c.36_39delACCT, p.(Tyr13*)	c.700C > T, p.(Arg234*)
20	*ALOXE3*	c.700C > T, p.(Arg234*)	c.700C > T, p.(Arg234*)
21	*ALOXE3*	c.700C > T, p.(Arg234*)	c.700C > T, p.(Arg234*)
22	*ALOXE3*	c.700C > T, p.(Arg234*)	c.700C > T, p.(Arg234*)
23	*ALOXE3*	c.700C > T, p.(Arg234*)	c.1889C > T, p.(Pro630Leu)
24	*ALOXE3*	c.700C > T, p.(Arg234*)	c.1889C > T, p.(Pro630Leu)
25	*ALOXE3*	**c.1392 + 2 T > A, r.spl?**	c.1889C > T, p.(Pro630Leu)
26	*ALOXE3*	c.1889C > T, p.(Pro630Leu)	c.2097C > T, p.(Tyr699*)
27	*ALOXE3*	c.1889C > T, p.(Pro630Leu)	gross deletion
28	*NIPAL4*	c.527C > A, p.(Ala176Asp)	c.527C > A, p.(Ala176Asp)
29	*NIPAL4*	c.527C > A, p.(Ala176Asp)	c.527C > A, p.(Ala176Asp)
30	*NIPAL4*	c.527C > A, p.(Ala176Asp)	**c.1010_1015dupTCAGCA, p.(Ser338_Thr339insIleSer)**
31	*NIPAL4*	c.527C > A, p.(Ala176Asp)	c.1193dupT, p.(Val401Argfs*36)
32	*NIPAL4*	c.1063delC, p.(Leu355Trpfs*93)	c.1063delC, p.(Leu355Trpfs*93)
33	*NIPAL4*	**c.1112C > G, p.(Ser371Leu)**	**c.1112C > G, p.(Ser371Leu)**
34	*CYP4F22*	**c.1A > G, (p. Met1?)**	**c.59dupG,** p.(Ile21Hisfs*59)
35	*CYP4F22*	c.59dupG, p.(Ile21Hisfs*59)	c.59dupG, p.(Ile21Hisfs*59)
36	*CYP4F22*	c.59dupG, p.(Ile21Hisfs*59)	c.59dupG, p.(Ile21Hisfs*59)
37	*CYP4F22*	**c.844C > T, p.(Arg282Trp)**	**c.1085G > A, p.(Arg362Gln)**
38	*CYP4F22*	**c.1085G > A, p.(Arg362Gln)**	**c.1085G > A, p.(Arg362Gln)**
39	*TGM1*	c.376C > T, p.(Arg126Cys)	c.919C > T, p.(Arg307Trp)
40	*TGM1*	c.377G > A, p.(Arg126His)	c.377G > A, p.(Arg126His)
41	*TGM1*	c.425G > A, p.(Arg142His)	**c.1184C > T p.(Thr395Ile)**
42	*TGM1*	c.425G > A, p.(Arg142His)	**c.2000 T > G, p.(Leu667Arg)**
43	*TGM1*	c.968G > A, p.(Arg323Gln)	c.1135G > C, p.(Val379Leu)
44	*TGM1*	**c.1310 T > G, p.(Val437Gly)**	**c.2307C > G, p.(Ser769Arg)**
45	*ABCA12*	**c.69G > A, p.(Pro23=), r.(spl?)**	c.5641C > T, p.(Arg1881*)
46	*ABCA12*	c.483_484delCGinsT, p.(Ala162Hisfs*10)	**c.4977G > A, p.(Glu1659=), r.(spl?)**
47	*ABCA12*	**c.2634C > G, p.(Phe878Leu)**	c.4139A > G, p.(Asn1380Ser)
48	*KRT1*	c.532 T > C, p.(Ser178Pro)^a^	–
49	*KRT1*	**c.593C > T, p.(Val198Gly)** ^**b**^	–
50	*KRT1*	c.1016delT, p.(Met339Argfs*23)	**–**
51	*KRT10*	c.467G > A, p.(Arg156His)^**b**^	**–**
52	*KRT10*	c.467G > A, p.(Arg156His)^**b**^	**–**
53	*KRT10*	c.1373 + 1G > C, r.spl?	**–**
54	*KRT10*	c.1374-1G > C, r.spl?	**–**
55	*KRT2*	c.1435A > C, p.(Thr479Pro)^c^	**–**
56	*KRT2*	c.1459G > A, p.(Glu487Lys)^c^	**–**
57	*STS*	**c.1330C > T, p.(His444Tyr)**	**–**
58	*STS*	**c.1338C > G, p.(Cys446Trp)**	–
59	*SPINK5*	**c.81 + 1G > A, r.spl?**	c.1431-12G > A, r.(spl?)

Variants in bold letters were detected only in Czech patients (31 patients were mentioned in our previous study [2]). Genes, reference sequences: *ALOX12B*, NM_001139.2; *ALOXE3*, NM_021628.2; *CYP4F22*, NM_173483.3; *NIPAL4*, NM_001099287.1; *TGM1*, NM_000359.2; *ABCA12*, NM_173076.2; *KRT1*, NM_006121.3; *KRT10*, NM_000421.3; *KRT2*, NM_000423.2; *STS*, NM_000351.5; *SPINK5*, NM_006846.3. The localisation of variants in a keratin molecule: ^a^the head domain, subdomain H1; ^b^the central rod domain, subdomain 1A, helix initiating motive; ^c^the central rod domain, subdomain 2A, helix terminating motif (www.interfil.org)

*ABCA12* disease-causing variants have been described in ARCI including harlequin ichthyosis (HI), congenital ichthyosiform erythroderma (CIE), and lamellar ichthyosis (LI) [3]. HI shows the most severe phenotype and most mutations associated with this phenotype create a premature termination codon (PTC). CIE and LI are clinically characterized by fine, whitish scales on a background of erythematous skin, and large, thick, dark scales over the entire body without a serious background erythroderma, respectively. We have three patients with pathogenic variants in *ABCA12*, two of them have a PTC mutation and a silent variant as a second mutation, but probably affecting mRNA splicing. The phenotype of both patients is very severe, including a picture harlequin foetus after birth; later (at 3 and 6 years of age, respectively) ectropin, eclabion, generalised large polygonal scaling and erythema (Fig. 1). To explore the possible association of the disease with the *ABCA12* gene, immunohistochemical ABCA12 protein analysis was performed in the case of patient 45. This analysis revealed deficient ABCA12 expression in the patient compared with normal skin tissue (Fig. 2). Patient 47, with two *ABCA12* missense mutations, has a milder phenotype with clinical findings corresponding to classical CIE.

**Fig. 1:**
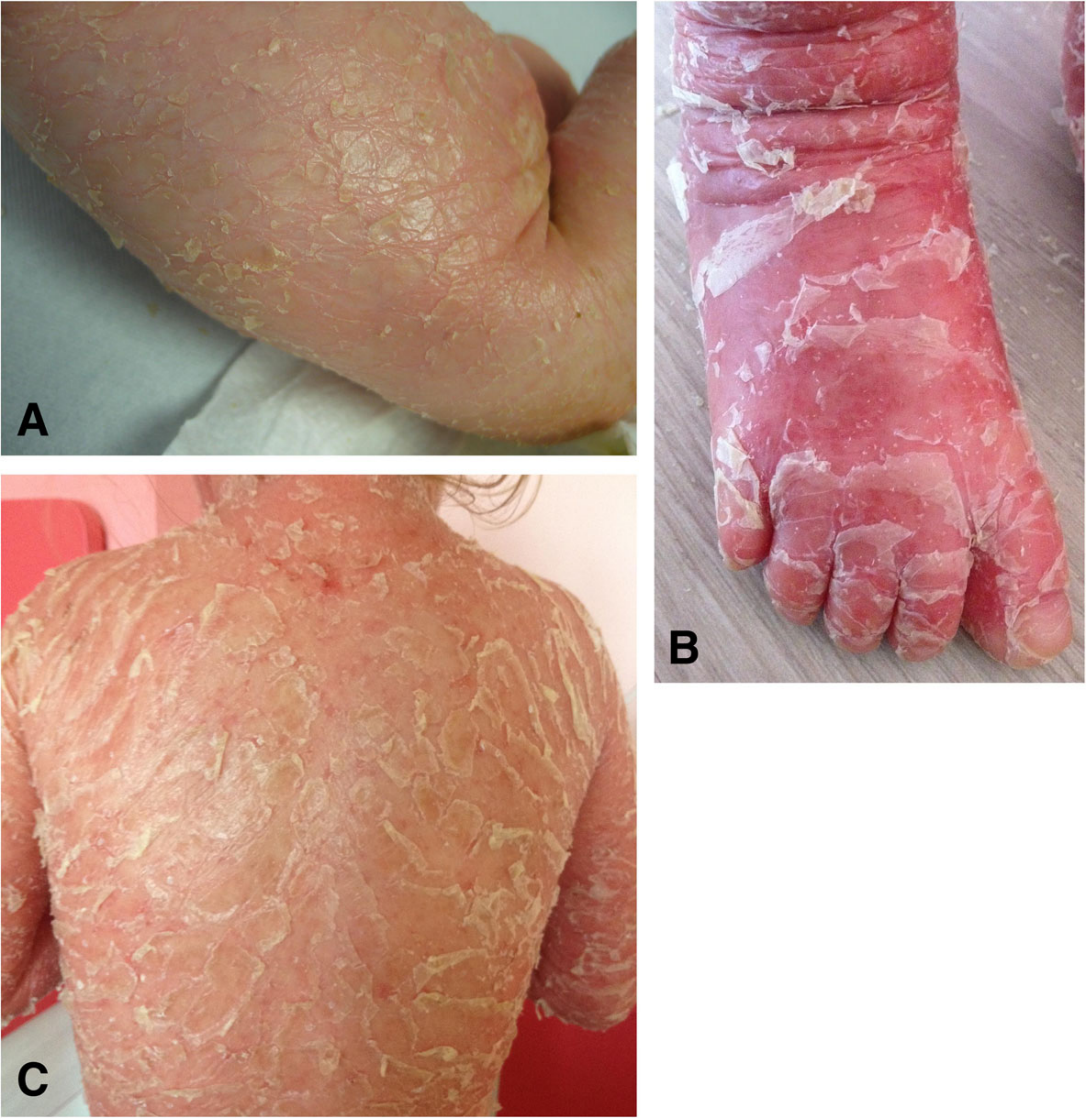
Photos of patient 45 at the age of 3 months (**a**) and 3 years (**b**, **c**)

**Fig. 2:**
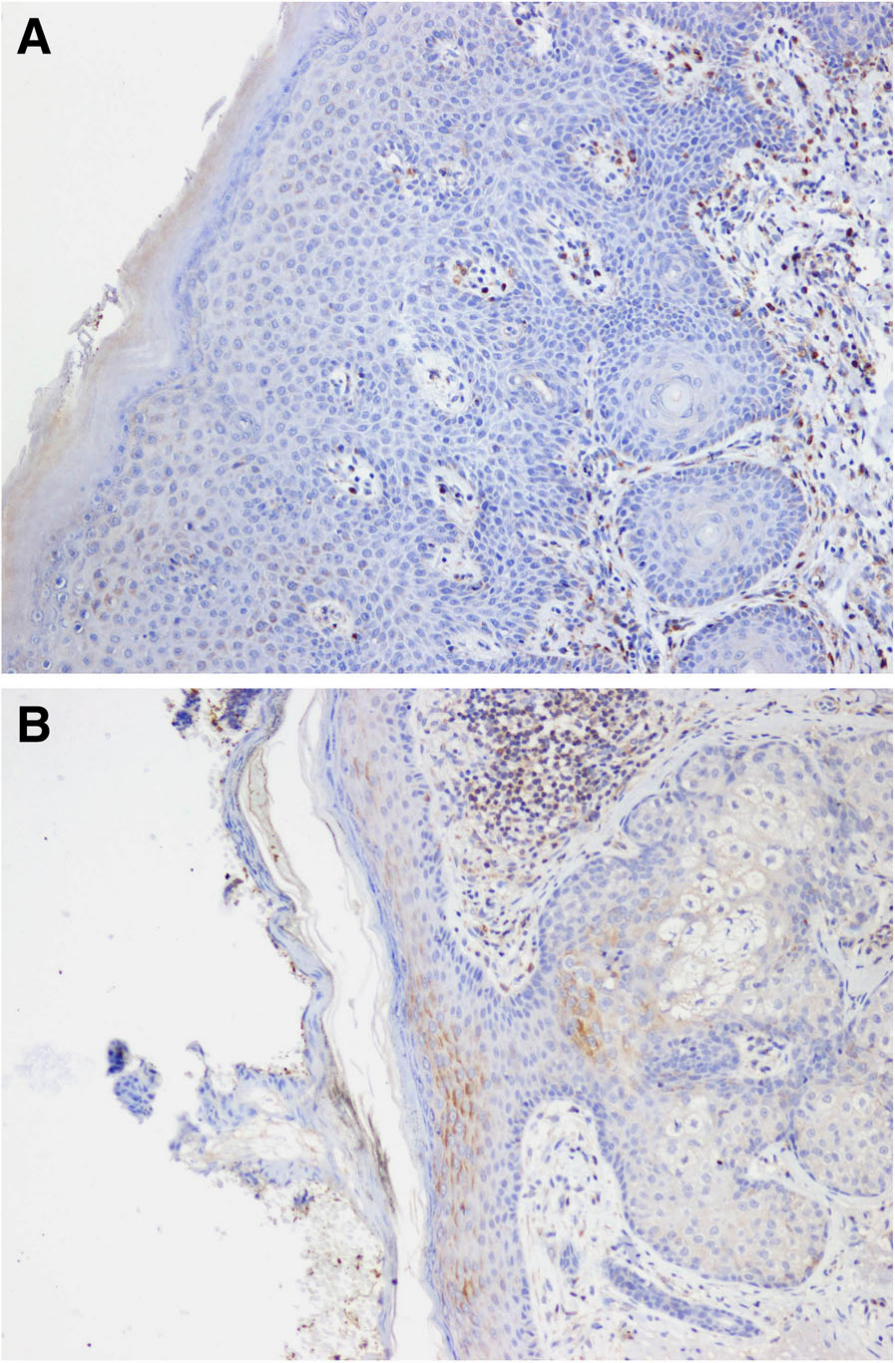
Immunohistochemical detection of the ABCA12 protein in skin tissue of the patient 45 (**a**) and a control (**b**), original magnification × 100

From 9 KI patients, 3 patients have a pathogenic sequence variant in *KRT1*, 4 patients in *KRT10*, and 2 patients in *KRT2*. Mutations in the mentioned *KRT* genes have autosomal dominant inheritance. Unfortunately, the parent’s DNA was not available in patients 48, 51, and 52 (but in all cases the identified variants were already described in HGMD as disease-causing). In patients 49, 53, and 56, a pathogenic variant was present in one of the parents in agreement with clinical symptoms. In patient 50, 54, and 55 pathogenic variants arose as a de novo event.

As multiple genes are linked to an ichthyosis phenotype, massive parallel sequencing is effective technique for molecular genetic diagnostics. This methodical approach generates a large amount of data which need to be interpreted from the point of view of their potential disease association. We performed in silico analysis of sequence variants based on 3D protein structures and commonly used prediction programs (such as SIFT, PolyPhen-2, and MutationTaster) to evaluate the effect of 76 pathogenic and 9 benign missense variants and compare the yields of these two approaches to each other. Suitable 3D protein structures from the Protein Data Bank (https://www.rcsb.org/) were found for the STS, ALOX12B, ALOXE3, and TGM1 proteins. Pathogenic sequence variants were either identified in our patients or reported in the Human Gene Mutation Database. Benign sequence variants were described in literature and/or indicated in the ExAC database (http://exac.broadinstitute.org) with an allele frequency > 1%. This strategy has been used in our previous studies [4–6].

Based on 3D protein structures, we were able to explain a deleterious effect of 74 pathogenic variants (97.4%). The structural defect of sequence variants was caused by a loss of structural contacts (i.e. direct H-bonds, salt bridges, stacking interactions), a change in physico-chemical properties, or their combinations. Considering benign variants, we observed that they are associated with no structural defects, and they are mostly localised on the protein’s surface. The structural analysis is described in more detail in Additional file 2. When we used SIFT, PolyPhen-2, and MutationTaster, the degree of compliance in prediction programs and phenotype status was 85.7% for pathogenic variants and 55.5% for benign variants, assuming that the results of all three prediction programs agree. Based on published recommendations [7], the combination of predictions from different prediction programs are considered as a single piece of evidence in sequence interpretation. If all of the prediction programs tested agree on the prediction, then this evidence can be counted as supporting. However, if predictions disagree, then this evidence should not be used in classifying a variant. Our results show that the detailed structural analysis of proteins is a better approach to interpret sequence variants (if an appropriate 3D protein structure is available) – the degree prediction compliance and phenotype status was 97.4% for pathogenic variants and 100% for benign variants (in contrast to 85.7% and 55.5% in commonly used prediction programs).

## Additional files

Additional file 1: **Table S1.** Patients’ clinical findings. (XLSX 23 kb)

Additional file 2: In silico analyses. Methods and results related to in silico analyses. (DOCX 71 kb)

## Abbreviations

ARCI: Autosomal recessive congenital ichthyosis
CIE: Congenital ichthyosiform erythroderma
HI: Harlequin ichthyosis
KI: Keratinopathic ichthyosis
LI: Lamellar ichthyosis
PTC: Premature termination codon
XLI: X-linked ichthyosis
