# Supplementary material for: Inherited ichthyoses: molecular causes of the disease in Czech patients
Source: Orphanet J Rare Dis. 2019 May 2;14:92. doi: 10.1186/s13023-019-1076-7 (PMC6498588; doi:10.1186/s13023-019-1076-7)
Supplement: Supplementary file 2 — In silico analyses. Methods and results related to in silico analyses. (DOCX 71 kb) [file 13023_2019_1076_MOESM2_ESM.docx]

***In silico* analyses**

**Methods**

***Building 3D protein structures***

We built 3D models for proteins coded by the *ALOX12B, ALOXE3,* and *TGM1* genes using homology modelling with the Modeller program [[1](#_ENREF_1)]. The X-ray structure of human 15-lipoxygenase-2 (PDB code: 4NRE) was used to model 12R-lipoxygenase (ALOX12B) consisting of 701 aminoacids (AAs) (sequence identity 48%) and lipoxygenase 3 (ALOXE3) consisting of 711 AAs (sequence identity 51%). Both proteins contain Fe^2+^ cation, its position was placed on the homology models based on its superposition with the template structure. In the *TGM1* gene coding transglutaminase-1, we used the human factor XIII structure (PDB code: 1EX0) that shares a 44% sequence identity to TGM1. However, for the terminal parts (AAs 1-80 and 791-817) there was no template structure, so these parts were not present in our model. As we do not know how these parts fold and interact with the rest of the protein, we analysed the impact of sequence variants only in the area, which should not be affected by the interaction with these missing parts (residues 320-790). TGM1 contains Ca^2+^ cation, which was placed on the homology models by superposition with the template structure.

For the steroid sulfatase coded by the *STS* gene, we used the human X-ray structure of estrone sulfatase (PDB code: 1P49; sequence identity 99%). This X-ray structure lacks the N-terminal region (AAs 1-22), so our model consisted only of residues 23-583. Further, a short sequence in the middle of the X-ray structure is missing (AAs 477-480), therefore sequence variants localised in this area were not analysed. Since the steroid sulfatase exists as a dimer, we built the dimeric structure based on the homologous protein N-acetylgalactosamine-6 sulfatase (PDB code: 4FDI) which was determined in the dimeric form. Each subunit of the STS protein contains one Ca^2+^ cation.

***Structural analysis of sequence variants***

Pathogenic sequence variants were either identified in our patients or reported in the Human Gene Mutation Database (HGMD). Benign sequence variants were described in literature and/or indicated in the ExAC database (exac.broadinstitute.org) as sequence variants with an allele frequency > 1%. In particular, we analysed benign sequence variants p.(Val55Met) and p.(Pro127Ser) in ALOX12B [[2](#_ENREF_2)]; p.(Leu237Met) [[2](#_ENREF_2)], p.(Ile515Val) [[3](#_ENREF_3)], p.(Arg670Trp) [[3](#_ENREF_3)], and p.(Arg678Cys) in ALOXE3; p.(Val518Met) [[4](#_ENREF_4)],[[5](#_ENREF_5)] in TGM1; and p.(Val307Ile) and p.(Val476Met) [[6](#_ENREF_6)] in STS.

The structural analysis was carried out only for substitutions in AA positions conserved in the template structure. Using the VMD program [[7](#_ENREF_7)], we analysed the side chain contacts of wild type (wt) AAs, i.e. direct H-bonds, salt bridges, and stacking interactions. In addition, we measured the buriedness of the wt AAs in the protein structure. The residues’ solvent accessibility in the protein structure was calculated using the STRIDE program (<webclu.bio.wzw.tum.de/cgi-bin/stride/stridecgi.py>) and divided by the total surface area of the residue [[8](#_ENREF_9)]. This value corresponds to the relative accessible surface area (RSA). A residue was considered buried if the RSA is ≤10 %. Replacing a buried AA is more likely to be associated with structural defects especially when volume, charge, and polarity change upon a mutation, and thus we measured these parameters for buried residues. Volume change upon a mutation was calculated [[9](#_ENREF_10)], a change ≥20 Å^3^ associated with the large to small substitution was considered destabilizing. A charge change upon a mutation was considered between charged and uncharged AAs and a polarity change was considered between nonpolar (Leu, Ile, Phe, Trp, Cys, Met, Val, Tyr), polar (Pro, Ala, Thr, Gly, Ser), and very polar (His, Arg, Gln, Lys, Asn, Glu, Asp) AAs. Further, we detected the replacement of wt proline and glycine residues in turns where they are key structural factors. In helices, any AA mutation to proline was considered destabilizing as proline is known to break a helical structure.

The impact of missense variants was also analysed using commonly used prediction programs such as SIFT, PolyPhen-2, and MutationTaster (Table A2). In the case of PolyPhen-2, we used the HumVar mutation model, which is recommended for Mendelian disease diagnostics.

**Results**

Structural analysis was performed for 76 pathogenic and 9 benign missense sequence variants (Table A2). In STS, 17 pathogenic (2 identified in the Czech patients and 15 reported in HGMD) and 2 benign variants were analysed. In ALOX12B, 11 pathogenic variants were identified, 9 of them (for which the wt AA is conserved in the template structure) were analysed together with 18 HGMD disease-causing variants and 2 benign variants. One ALOXE3 pathogenic variant was detected, this was analysed together with 4 HGMD disease-causing variants and 4 benign variants. Ten TGM1 pathogenic variants were determined, 3 of them (with conserved wt AA in the template structure) were evaluated together with 24 HGMD disease-causing variants and 1 benign variant. Using structural analysis, we were able to explain the causality of 74 variants (97.4%). The structural defect is either caused by a loss of structural contact [e.g. p.(Lys222Ile) and p.(Arg386His) in ALOX12B], or a change in physico-chemical properties [e.g. p.(Pro630Leu) and p.(Gly281Val) in ALOXE3] or their combination [e.g. p.(Glu394Lys) in ALOX12B and p.(Ser769Arg) in TGM1]. Considering benign variants in the ALOX12B, ALOXE3, TGM1 and STS proteins, we observed that they are associated with no structural defects, and they are mostly localised on the protein’s surface. Using molecular modelling, we did not find any defects for two variants, p.(Ala560Gly) and p.(Leu717Arg) in TGM1, which could explain their pathogenicity. This might be elucidated by either insufficiently strict structural criteria in our analysis or an imperfect TGM1 homology model, which we used as a template with lower sequence identity (44%). In addition, two longer regions in the TGM1 protein were missing, hence the fold arrangement of this protein was not fully complete.

Except for the variants p.(Tyr521Cys) in *ALOX12B* and p.(Ala176Asp) in *NIPAL4*, frequently occurring variants (observed at least 3 times) reside either on the CpG dinucleotide (e.g. p.(Ala597Glu) in *ALOX12B*, p.(Arg234*) in *ALOXE3*, and p.(Arg362Gln) in *CYP4F22*) or CpHpG trinucleotide (where H stands for A, C or T) (e.g. p.(Pro630Leu) in *ALOXE3*) [[10](#_ENREF_11)].

**References**

[1] Sali A, Blundell TL: Comparative protein modelling by satisfaction of spatial restraints. J Mol Biol 234 (1993) 779-815.

[2] Eckl KM, Krieg P, Kuster W, Traupe H, Andre F, Wittstruck N, et al.: Mutation spectrum and functional analysis of epidermis-type lipoxygenases in patients with autosomal recessive congenital ichthyosis. Hum Mutat 26 (2005) 351-361.

[3] Lesueur F, Bouadjar B, Lefevre C, Jobard F, Audebert S, Lakhdar H, et al.: Novel mutations in ALOX12B in patients with autosomal recessive congenital ichthyosis and evidence for genetic heterogeneity on chromosome 17p13. J Invest Dermatol 127 (2007) 829-834.

[4] Herman ML, Farasat S, Steinbach PJ, Wei MH, Toure O, Fleckman P, et al.: Transglutaminase-1 gene mutations in autosomal recessive congenital ichthyosis: summary of mutations (including 23 novel) and modeling of TGase-1. Hum Mutat 30 (2009) 537-547.

[5] Pigg MH, Bygum A, Ganemo A, Virtanen M, Brandrup F, Zimmer AD, et al.: Spectrum of Autosomal Recessive Congenital Ichthyosis in Scandinavia: Clinical Characteristics and Novel and Recurrent Mutations in 132 Patients. Acta Derm Venereol 96 (2016) 932-937.

[6] Matsumoto J, Ariyoshi N, Ishii I, Kitada M: Functional characterization of seven single-nucleotide polymorphisms of the steroid sulfatase gene found in a Japanese population. J Hum Genet 58 (2013) 267-272.

[7] Humphrey W, Dalke A, Schulten K: VMD: visual molecular dynamics. J Mol Graph 14 (1996) 27-38.

[8] Chothia C: The nature of the accessible and buried surfaces in proteins. J Mol Biol 105 (1976) 1-12.

[9] Zamyatin AA: Protein Volume in Solution. Prog Biophys Mol Biol 24 (1972) 107-123.

[10] Cooper DN, Mort M, Stenson PD, Ball EV, Chuzhanova NA: Methylation-mediated deamination of 5-methylcytosine appears to give rise to mutations causing human inherited disease in CpNpG trinucleotides, as well as in CpG dinucleotides. Hum Genomics 4 (2010) 406-410.

**Table A2.** Effects of ALOX12B, ALOXE3, TGM1, and STS missense sequence variants on the protein structure

| Sequence variant | Wt  side chain  contacts | Buriedness  of wt AA  (RSA in %) | Charge change | Polarity change | Volume  change (Å^3^) | Pro/Gly  change | SIFT/  Polyphen2/  Mutation Taster |
| --- | --- | --- | --- | --- | --- | --- | --- |
| **ALOX12B disease-causing sequence variants** | | | | | | | |
| **p.(Lys222Ile)** | Asp228 | 22 |  |  |  |  | **T**/**B**/**P** |
| **p.(Gln357His)** | Ser359,  Gln360 | 0 |  |  |  |  | D/PrD/DC |
| **p.(Arg386His)** | Asn594 | 4 |  |  |  |  | D/PrD/DC |
| **p.(Arg386Cys)** | Asn594 | 4 |  |  |  |  | D/PrD/DC |
| p.(Pro422Leu) |  | 2 |  |  | 54 |  | D/PrD/DC |
| **p.(Arg469Trp)** | Glu322 | 43 |  |  |  |  | D/PrD/**P** |
| **p.(Arg499His)** | Val490, Leu493,  Asp500 | 19 |  |  |  |  | D/(PrD/PrD)/DC |
| p.(Tyr521Cys) | Asp530 | 2 |  |  |  |  | D/PrD/DC |
| **p.(Leu563Pro)** |  | 0 |  |  |  |  | D/PoD/DC |
| p.(Gln360Glu)***** | Arg319 | 5 |  |  |  |  | D/PrD/DC |
| p.(Lys382Glu)***** | Asp261,  Ala259 | 3 |  |  |  |  | D/PrD/DC |
| p.(Thr383Met)***** |  | 2 |  |  | 47 |  | **T/**PoD/DC |
| p.(Glu394Lys)***** | His398 | 8 |  |  |  |  | **T**/PoD/DC |
| p.(His398Tyr)***** | Glu394, Fe ion | 2 |  |  | 40 |  | D/PrD/DC |
| p.(His403Tyr)***** | Fe ion | 7 |  |  | 40 |  | D/PrD/DC |
| p.(His421Tyr)***** | Asp530 | 0 |  |  | 40 |  | D/PrD/DC |
| p.(Leu426Pro)***** |  | 2 |  |  |  |  | D/PrD/DC |
| p.(Arg442Trp)***** | Glu407, Ile188 | 3 |  |  | 54 |  | D/PrD/DC |
| p.(Gly462Asp)***** |  | 0 |  |  | 51 |  | D/PrD/DC |
| p.(Gly462Ser)***** |  | 0 |  |  | 29 |  | **T/**PrD/DC |
| p.(Arg488His)***** | Tyr496, Gly275 | 6 |  |  |  |  | D/PrD/DC |
| p.(Val527Met)***** |  | 0 |  |  | 23 |  | D/PrD/DC |
| p.(Glu532Gln)***** | Arg133 | 13 |  |  |  |  | D/PrD/DC |
| p.(His578Gln)***** | Fe ion | 4 |  |  |  |  | D/PrD/DC |
| p.(Lys607Asn)***** | Asp373 | 36 |  |  |  |  | D/PrD/DC |
| p.(Arg679Leu)***** | Asp501 salt bridge | 19 |  |  |  |  | D/PrD/DC |
| p.(Arg679Ser)***** | Asp501 salt bridge | 19 |  |  |  |  | D/PrD/DC |
| **ALOX12B benign sequence variants** | | | | | | | |
| p.(Val55Met) |  | 51 |  |  |  |  | **D/PoD**/P |
| p.(Pro127Ser) |  | 65 |  |  |  |  | T/B/P |
| **ALOXE3 disease-causing sequence variants** | | | | | | | |
| p.(Pro630Leu) |  | 0 |  |  | 54 |  | D/PrD/DC |
| p.(Gly281Val)***** |  | 10 |  |  | 80 |  | D/PrD/DC |
| p.(Arg396Ser)***** | Glu399, Gln277 | 3 |  |  |  |  | D/PrD/DC |
| p.(Leu427Pro)***** |  | 0 |  |  |  |  | D/PrD/DC |
| p.(Val500Phe)***** |  | 0 |  |  | 50 |  | D/PrD/DC |
| **ALOXE3 benign sequence variants** | | | | | | | |
| p.(Leu237Met) |  | 9 |  |  |  |  | T/**PoD**/P |
| p.(Ile515Val) |  | 0 |  |  |  |  | T/B/P |
| p.(Arg670Trp) |  | 74 |  |  |  |  | **D**/B/P |
| p.(Arg678Cys) |  | 32 |  |  |  |  | T/B/P |
| **TGM1 disease-causing sequence variants** | | | | | | | |
| p.(Arg323Gln) | Asp490,  Gly473 | 31 |  |  |  |  | D/PrD/DC |
| p.(Val379Leu) |  | 0 |  |  | 27 |  | **T/B**/DC |
| p.(Ser769Arg) | Ala354 | 0 |  |  | 84 |  | **T/**PrD/DC |
| p.(Val383Met)* |  | 0 |  |  | 23 |  | D/PrD/DC |
| p.(Leu388Pro)* |  | 4 |  |  |  |  | D/PrD/DC |
| p.(Arg389His)* | Asn270, Thr395 | 3 |  |  |  |  | D/PrD/DC |
| p.(Gly392Asp)* |  | 0 |  |  | 51 |  | D/PrD/DC |
| p.(Arg396His)* | Asp440 | 6 |  |  |  |  | D/PrD/DC |
| p.(Arg396Leu)* | Asp440 | 6 |  |  |  |  | D/PrD/DC |
| p.(Arg396Ser)* | Asp440 | 6 |  |  |  |  | D/PrD/DC |
| p.(His405Asn)* | Pro462, Glu497 | 5 |  |  |  |  | D/PrD/DC |
| p.(Asp414Val)* | Tyr416 | 20 |  |  |  |  | D/PrD/DC |
| p.(Asp430Val)* | Leu411 | 51 |  |  |  |  | D/PrD/DC |
| p.(His436Asp)* | Asp459 | 0 |  |  |  |  | D/PrD/DC |
| p.(Gly451Val)* |  | 62 |  |  |  |  | D/PrD/DC |
| p.(Trp455Arg)* |  | 8 |  |  |  |  | D/PrD/DC |
| p.(Gln463His)* |  | 4 |  |  |  |  | D/PrD/DC |
| p.(Gly473Ser)* |  | 0 |  |  | 29 |  | D/PrD/DC |
| p.(Ile480Phe)* |  | 0 |  |  | 23 |  | D**/B**/DC |
| p.(Asp490Gly)* | Arg323, Tht465 | 21 |  |  |  |  | D/PrD/DC |
| p.(Gly524Asp)* |  | 3 |  |  | 51 |  | D/PrD/DC |
| p.(Gly524Ser)* |  | 3 |  |  | 29 |  | D/PrD/DC |
| p.(Thr529Ile)* | Thr397 | 2 |  |  | 51 |  | D/PrD/DC |
| p.(Tyr544Cys)* | Asp440 | 0 |  |  |  |  | D/PrD/DC |
| p.(Ala560Gly)* |  | 0 |  |  |  |  | D/PrD/DC |
| p.(Leu717Arg)* |  | 14 |  |  |  |  | D/PrD/DC |
| p.(Arg764Cys)* | Asp208 | 7 |  |  |  |  | D/PrD/DC |
| **TGM1 benign sequence variants** | | | | | | | |
| p.(Val518Met) |  | 23 |  |  |  |  | T/**PoD**/**DC** |
| **STS disease causing sequence variants** | | | | | | | |
| **p.(His444Tyr)** | Arg454 | 3 |  |  | 40 |  | **T**/PrD/DC |
| p.(Cys446Trp) | 489Cys-SS bond | 2 |  |  | 119 |  | D/PrD/DC |
| p.(Pro151Arg)* |  | 0 |  |  | 61 |  | D/PrD/**P** |
| p.(Thr165Ile)* | K134 | 0 |  |  | 51 |  | D/PrD/DC |
| p.(Ser341Leu)* | Ala34, Leu37 | 0 |  |  | 78 |  | D/PrD/DC |
| p.(Gly359Arg)* |  | 23 |  |  | 113 |  | D/PrD/DC |
| p.(Gly367Arg)* |  | 0 |  |  | 113 |  | D/PrD/DC |
| p.(Trp372Arg)* |  | 10 |  |  |  |  | D/PrD/DC |
| p.(Trp372Ser)* |  | 10 |  |  |  |  | **T**/PrD/DC |
| p.(Gly380Arg)* |  | 0 |  |  | 113 |  | D/PrD/DC |
| p.(His444Arg)* | Arg454 | 3 |  |  |  |  | D/PrD/DC |
| p.(Cys446Tyr)* | 489Cys-SS bond | 2 |  |  | 85 |  | D/PrD/DC |
| p.(Arg454Cys)* | His444 | 2 |  |  |  |  | D/PrD/DC |
| p.(Arg454His)* | His444 | 2 |  |  |  |  | D/PrD/DC |
| p.(Lys465Glu)* | G373, Glu511 | 0 |  |  |  |  | D/PrD/DC |
| p.(Gln560Arg)* | Leu240 | 3 |  |  | 30 |  | D/PrD/DC |
| p.(Gln560Pro)* | Leu240 | 3 |  |  |  |  | D/PrD/DC |
| **STS benign sequence variants** | | | | | | | |
| p.(Val307Ile) |  | 31 |  |  |  |  | T/B/P |
| p.(Val476Met) |  | 58 |  |  |  |  | T/B/P |

T: tolerated, D: damaging, PrD: probably damaging, PoD: possibly damaging, P: polymorphism, DC: disease causing, B: benign; prediction not in agreement with patient´s phenotype is in bold. Grey fields show detected features based on 3D modelling. Disease-causing sequence variants identified only in Czech ARCI patients are in bold letters.

*Disease-causing sequence changes reported in HMGD.
